# Patient preference in allergen immunotherapy - Understanding the patient's view^☆^

**DOI:** 10.1016/j.waojou.2025.101154

**Published:** 2025-12-13

**Authors:** Sven Becker, Martin Feindor, Anke Graessel, Isabel Fernández de Alba, Katrin Birkholz, Jennifer Raab, Filippo Fassio, Dolores Hernandez, Neil Valentine, Robin Abro, Oliver Fuchs, Simon Blank, Markus Ollert, David Gonzalez-de-Olano, Ludger Klimek, Erika Jensen-Jarolim, Peter Schmid-Grendelmeier, Gerald Hofer, Matthias F. Kramer

**Affiliations:** a Department of Otorhinolaryngology, Head and Neck Surgery, University of Tübingen, Tübingen, Germany; b Bencard Allergie GmbH, Munich, Germany; c Allergy Therapeutics, Worthing, United Kingdom; d Hospital Universitario HLA Inmaculada, Granada, Spain; e SOSD Allergologia e Immunologica Clinica, Ospedale San Giovanni di Dio, Firenze - Azienda USL Toscana Centro, Italy; f Allergy Therapeutics Ibérica, Barcelona, Spain; g Lumanity, London, United Kingdom; h Allergology, Department of Medicine, Lucerne Cantonal Hospital and University of Lucerne, Lucerne, Switzerland; i Center of Allergy & Environment (ZAUM), Technical University of Munich, School of Medicine and Health & Helmholtz Munich, German Research Center for Environmental Health, Munich, Germany; j Department of Infection and Immunity, Luxembourg Institute of Health, Esch-sur-Alzette, Luxembourg; k Department of Dermatology and Allergy Centre, Odense Research Centre for Anaphylaxis, Odense University Hospital, University of Southern Denmark, Odense, Denmark; l Allergy Department, Hospital Universitario Ramon y Cajal, IRYCIS, Madrid, Spain; m Center for Rhinology and Allergology, Wiesbaden, Germany; n AllergyCare - Allergy Diagnosis Center, Private Clinic Döbling, Vienna, Austria; o Institute for Pathophysiology and Allergy Research, Medical University Vienna, Vienna, Austria; p Allergy Unit, Department of Dermatology, University Hospital of Zürich, Zurich, Switzerland

**Keywords:** Venom immunotherapy, Sublingual immunotherapy, Subcutaneous immunotherapy, Shared decision-making, Discrete choice experiment, Adherence

## Abstract

**Background:**

Allergen Immunotherapy (AIT) is an effective treatment for patients with pollen, house dust mite, or venom allergy, but treatment adherence can be challenging. Patient preferences play a crucial role in acceptance and adherence to AIT, but little is known about these preferences. This study aimed to understand patient preferences for AIT and how these preferences influence treatment acceptance.

**Methods:**

A conjoint analysis was conducted among 750 participants from 7 European countries who were allergic to pollen (n = 700) or Hymenoptera venom (n = 50) and had not previously received AIT. Participants were asked to choose between hypothetical AIT products with different attributes, including product type, initial up-dosing posology, potential future risks, and side effects. The relative importance of each attribute was calculated, and sensitivity analyses were performed to assess the impact of specific attribute levels on patient preference.

**Results:**

Potential future risk is the attribute with the strongest impact on the importance score for patient preference in both pollen (44%) and venom (41%) allergic patients, followed by side effects (24% for pollen and 35% for venom allergy). Product type was less important, with a 22% importance score in both populations, and dosing schedules were not important at all, with a 2% importance score for pollen and an 11% importance score for venom-allergic patients. Accumulation of foreign material/substance in the body had the largest negative impact on patient preference, with drops of −24.7% (pollen) and −23.6% (venom), respectively.

**Conclusions:**

Understanding patient preferences is essential for optimizing the design and delivery of AIT. Different side effects and risk profiles of AIT products can influence patient treatment acceptance the most, and healthcare professionals may not always be aware of it. Future research should focus on developing AIT products that align with patient preferences with simultaneously very high effectiveness to improve adherence and treatment outcomes.

## Graphical abstract

**Figure:**
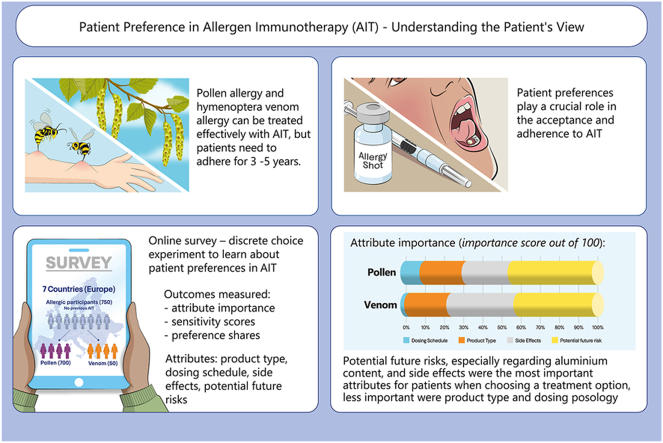

## Introduction

Patient-centered care and patient empowerment play a critical role in modern everyday medical practice.^1^ Incorporating patients' preferences fitting their lifestyle and respecting their private opinion on therapy options could facilitate a more collaborative interaction between physicians and patients and has the potential power to improve adherence to a specific therapy,^2^, ^3^, ^4^ more so than, for example, the severity of chronic diseases.^3^ Patients' preferences were one of the central motifs of Sackett's original concept of Evidence-Based Medicine (EBM).^5^ Shared Decision-Making (SDM), integrating patient preference into choice of treatment options, has become more commonly used.^6^ Allergen immunotherapy (AIT), being the only causative treatment option for patients suffering from allergic diseases such as allergic rhinitis due to airborne allergens (pollen, house dust mite, moulds, etc.) or insect venom hypersensitivity due to insect stings, is a type of therapy which demands a very high adherence from patients.^7^ Therefore, the process of SDM needs to be prioritized in patients suitable for allergen immunotherapy^8^ to potentially increase adherence.^9^

The 2 most frequently used application routes for pollen allergen immunotherapy are subcutaneous immunotherapy (SCIT) and sublingual immunotherapy (SLIT).^10^^,^^11^ Previous studies on patient preference in AIT for inhalant allergens have shown a tendency towards a profile best fulfilled by SLIT.^12^, ^13^, ^14^ This preference appears to be driven mainly by scheduling convenience, and safety concerns related to anaphylactic reactions.^13^

SCIT products are generally available on European markets as aqueous native allergens, alum-adjuvanted native allergens, alum-adjuvanted allergoids, MicroCrystalline Tyrosine (MCT)-adjuvanted allergoids or allergoids adjuvanted with the adjuvant system composed of MCT and Monophosphoryl Lipid-A (MPL).^10^ SLIT products available as tablets or drops are composed of non-adjuvanted native allergen extracts or allergoids.^10^

Venom immunotherapy (VIT) is a highly effective and well-tolerated treatment option for patients suffering from Hymenoptera venom allergies^15^ and is only available as SCIT, either aqueous extract or alum-adjuvanted.^16^^,^^17^ VIT has been shown to drastically decrease the risk of systemic reactions to Hymenoptera stings,^15^ as well as provide better quality of life.^18^ Even though adherence to VIT is generally high, there is still room for improvement.^19^ No studies have researched product-related preference related to venom immunotherapy (VIT) so far. Effective SCIT and SLIT regimens are also available against house dust mite allergy,^20^^,^^21^ but were not the focus of this study.

SCIT products can be split into aqueous and depot formulations, with the latter being primarily represented by aluminium hydroxide. Aluminium salts are the most used depot adjuvants in SCIT.^22^ Despite ongoing concerns,^23^ its benefit-risk ratio is considered acceptable.^22^ Nevertheless, the German Federal Institute for Vaccines and Biomedicines (Paul-Ehrlich-Institute, PEI) recently initiated a project to establish physiology-based toxicokinetic modelling of aluminium exposure from adjuvants in medicinal products.^24^, ^25^, ^26^, ^27^ In a recent small cross-sectional case-control study, Hiller et al^28^ found elevated aluminium excretion in urine samples given 24h after SCIT injection compared to before. Even though aluminium exposure is a topic of ongoing debate, its influence on patient preferences in AIT treatment choice has so far not been explored.

Depending on the formulation of the products, patients can be offered different dosing options and schedules, as well as different risk and tolerability profiles,^10^^,^^17^^,^^29^ all of which are likely to influence treatment acceptance. Studies have shown that respecting patient preference in treatment selection can significantly improve adherence and persistence,^3^^,^^4^^,^^9^ and, more importantly, influence the patient's decision to start treatment in the first place,^2^ applying the principle of shared decision-making.^8^ Therefore, this discrete-choice-experiment, as recommended by regulatory institutions,^30^ was set up to investigate patient preferences towards possible attributes of different AIT product profiles in pollen and venom allergy.

## Materials and methods

**Fig. 1:**
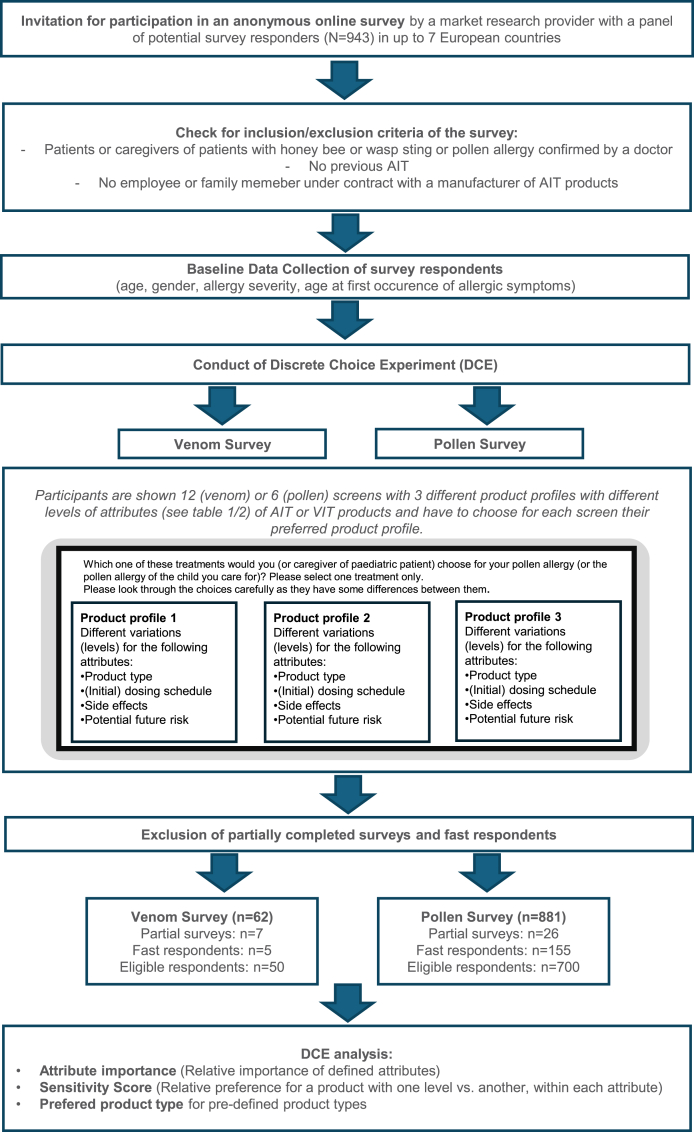
Study flowchart describing the general procedure of the DCE analysis

Within 1 project based on 2 cohorts, 1 for pollen patients and 1 for venom patients, the preference for a specific pollen AIT or VIT product profile was analysed using a 15-min online survey (full questionnaire in supplemental material). Pollen or Hymenoptera venom allergic adults (18–55 of age) and caregivers (18 years and older) as representatives of allergic children or adolescents (1–17 of age) from the European countries Germany, Austria, Switzerland, and Spain for the pollen part and Germany, Austria, Switzerland, United Kingdom, Italy, France, and Spain for the venom part were invited to participate in the anonymous online survey. Lumanity, a market research fieldwork panel provider with a European panel for online surveys, was used to distribute the survey to a pre-existing pool of potential survey participants on behalf of Allergy Therapeutics. The name of the sponsor was not disclosed to the participants to avoid possible bias. No pre-determined selection criteria were used for survey invites. The invites were distributed randomly across the respondent panel. The process of the analysis is depicted in Fig. 1.

The participation was voluntary and could be withdrawn at any time. Patients received an expense allowance for their participation. The survey was done in accordance with the EphMRA,^31^ ESOMAR,^32^ and MRS^33^ codes of conduct regarding anonymity and confidentiality. European Data Protection Law was followed, and only anonymized aggregated results were provided.

Participation was voluntary and could be withdrawn at any time. As a first step, patients gave their consent to the data protection declaration and survey participation, as well as agreement to evaluation and publication of the results (see questionnaire, supplemental material 1). The survey was conducted anonymously, and no ethical approval was required.

Data were collected from January to February 2023. Participation in the online survey was possible if the adult or child/adolescent suffered from an allergy to bee and/or wasp stings or pollen (grass, tree, ragweed, other) allergy. The respondents had to confirm that the allergy was confirmed or diagnosed by a physician, otherwise they could not take part in the survey. Adults or children/adolescents who were already treated with AIT or have a potential conflict of interest were not eligible to participate in the online survey.

The survey included questions assessing the respondent's (or the child/adolescent's) age, gender, allergy symptoms, frequency of allergy symptoms (pollen cohort), allergy severity grade, allergy treatments, annual household income and age of first occurrence of allergic symptoms. More detailed medical data points likely unavailable to most respondents, like immunological markers or other diagnostic criteria, were not included to avoid raising the barrier of entry. The respondents were separated into pollen and venom cohorts. To assess the preference for pollen or venom allergy treatment product profiles, a Discrete Choice Experiment (DCE) design was used.

### Conduct of DCE

The discrete-choice approach is explicitly recommended and acknowledged by regulatory institutions for the measurement of patient preferences.^30^ It is a standard preference method where participants are presented with different alternatives such as product profiles that comprise different attributes and attribute variations. The attributes and values for both cohorts are depicted in Table 1 and Table 2. The respondents have to choose the product profile that maximizes their utility. The respondents' relative preference can be analysed based on how they choose between different alternatives.^34^^,^^35^

**Table 1:** Predefined attributes and attribute variations (levels) for pollen AIT cohort

Attribute	Attribute variations (levels)		
	1	2	3
Product type	A tablet placed under the tongue for at least 1 min, and then swallowed as it dissolves	An injection not containing aluminium, taken in the upper arm	An injection containing aluminium, taken in the upper arm
Initial up-dosing schedule	Everyday for 3 years taken at home	6 times a year for 3 consecutive years at doctor's office or clinic/hospital before pollen season starts	Monthly for 3 consecutive years at doctor's office or clinic/hospital
Side effects	Pain and/or swelling in the mouth/throat [SHOW IF TABLET]/in arm [SHOW IF INJECTION]	Lumps at injection site (may persist for months/years and can be tender/itchy/have a bad appearance)^1^, ^2^, ^3^, ^4^, ^5^, ^6^	
Potential future risk^a^	None	Being excluded from future treatments/vaccines containing same ingredient as this treatment^31^	Accumulation of foreign material/substance in the body

a Affecting a small percentage of patients.

**Table 2:** Predefined attributes and attribute variations (levels) for venom cohort

Attribute	Attribute variations (levels)		
	1	2	3
Product type	An injection not containing aluminium, taken in the upper arm	An injection containing aluminium, taken in the upper arm	An injection containing aluminium, taken in the upper arm
Initial up-dosing schedule	Administered for 2, 3, or 5 consecutive days requiring surveillance in hospital *After initial dose schedule, dosing is monthly for at least 3 years, throughout the year at hospital/clinic or doctor's office*	Administered weekly for 15–16 weeks by a healthcare professional *After initial dose schedule, dosing is monthly for at least 3 years, throughout the year at hospital/clinic or doctor's office*	Administered weekly for 25 weeks by a healthcare professional *After initial dose schedule, dosing is monthly for at least 3 years, throughout the year at a hospital/clinic or doctor's office*
Side effects	Pain and/or swelling in the arm	Lumps at injection site (may persist for months/years and can be tender/itchy/have a bad appearance)^1^, ^2^, ^3^, ^4^, ^5^, ^6^	Lumps at injection site (may persist for months/years and can be tender/itchy/have a bad appearance)^1^, ^2^, ^3^, ^4^, ^5^, ^6^
Potential future risk^a^	None	Being excluded from future treatments/vaccines containing same ingredient as this treatment^31^	Accumulation of foreign material/substance in the body

a Affecting a small percentage of patients.

Marketed pollen AIT and VIT products share many similarities, so we focused on differentiators potentially relevant to patient's preferences such as posology- or composition-related factors. To analyze the patient preference for the attributes of potential pollen AIT and VIT products, we defined the attributes of product type, (initial) dosing schedule, side effects, and potential future risks as well as the corresponding attributes variations/levels. The options were presented under the assumption of equal efficacy. The attributes and levels are depicted in Table 1 for venom and Table 2 for pollen AIT.

Pollen AIT is available as subcutaneous or sublingual allergen immunotherapy. To address the preference, the product types of: i) tablets, ii) injections containing aluminium, and iii) injections not containing aluminium were defined as different levels of product types. Furthermore, pollen AIT products do differ in their dosing schedules. To analyze the patient's preference for the dosing schedule, the following 3 different levels were defined: i) every day for 3 years taken at home (ie, SLIT), ii) 6 times a year for 3 consecutive years at doctor's office or clinic/hospital before pollen season starts (ie, short-course SCIT), or iii) monthly for 3 consecutive years at doctor's office or clinic/hospital (ie, perennial SCIT).

VIT products differ in their initial dosing schedule to reach maintenance dose where monthly injections are performed for at least 3 years. To examine the preference for patients to different up-dosing schedules in accordance with manufacturer's recommended posology, the levels: i) administered for 2, 3, or 5 consecutive days requiring surveillance in hospital (ie, Rush/Ultra-Rush posology), ii) administered weekly for 15–16 weeks by a healthcare professional, and iii) administered weekly for 25 weeks by a healthcare professional (ie, conventional posology) were defined.

Another important factor for patients' preference and adherence are side effects and potential future risks of therapies. Therefore, known safety concerns should be discussed before initiating an AIT.^10^ Main side effects of allergen immunotherapy are related to the fact that the patient is allergic to the allergen administered. This is considered a class effect, and no differences exist in systemic reaction profiles between marketed products.^36^ The main differentiator between aqueous extracts and almost all depot formulations is the use of aluminium adjuvants. There are certain consequences that can influence the patient's preference. Among those consequences applicable for aluminium depot formulations are:

• injection of foreign material with the potential for long-lasting accumulation within the body.^22^^,^^37^^,^^38^
• granuloma formation, potentially presenting as persisting itching nodules at the injection site with excoriations, hyperpigmentation, and/or hypertrichosis.^39^^,^^40^
• and induction of aluminium-induced delayed hypersensitivity^22^ with the consequence of having to avoid future aluminium adjuvanted vaccinations or SCIT.

As for most drugs, allergy against 1 of the ingredients constitutes an exclusion criterion for future use.^41^ This is particularly true for adjuvants used in subcutaneous AIT and vaccination against infections. Aluminium in vaccines and preparations for AIT is the major sensitization source^39^ for aluminium contact allergy. Being excluded from future aluminium-adjuvanted vaccines might constitute a consequence of an aluminium hypersensitivity induced by depot pollen AIT or VIT.

To address these aspects, the attributes “side effects” and “potential future risks” on patient preference were analysed in this survey. For “side effects”, the attribute levels i) Pain and/or swelling in the mouth/throat (show if tablet)/in arm (show if injection) and ii) Lumps at the injection site (may persist for months/years and can be tender/itchy/have a bad appearance) were defined. Although a potential adverse effect occurs only in a minor number of patients, we addressed in this survey a possible impact on patients’ preference if a product bears the potential risk of being excluded from future treatments/vaccinations containing the same adjuvant as the theoretical pollen AIT or VIT profile.

The levels from each attribute can be combined to form multiple product profiles. The full factorial choice design would lead to 54 possible product profiles for the pollen AIT cohort or 36 possible product profiles for the venom cohort. In line with common practice,^35^ we developed a fractional-factorial design with restrictions on disallowed combinations using Sawtooth's CBC software.

All eligible participants were shown 6 (pollen) or 12 (venom) screens with 3 different product profiles and they marked their preferred product profile (see Fig. 1). A pilot survey was conducted with 9 participants to ensure the language and structure were clear, with no issues identified.

The conjoint analysis produces the following outputs:

- Attribute importance: This describes the relative importance of the defined attributes in relation to each other; it is defined as the relative share of each attribute's utility range (difference between most and least preferred levels), compared to the total utility ranges across all attributes. It is expressed as a percentage out of 100%.
- Sensitivity scores: These describe how strongly changes in an attribute's level (ie, product characteristics) affect choice probability; they are defined as the magnitude of the change in preference towards an option caused by the change in level. They are expressed as a percentage delta. A sensitivity score above ± 10% is generally regarded as having at least moderate influence on choice preference.
- Preference shares for pre-defined product types: Using the previous outputs, the expected preference towards a fixed combination of attribute levels (see Table 3 for pollen AIT cohort and Table 4 for venom cohort) can be derived and expressed as a percentage out of 100% between 2 or more choices.

**Table 3:** Pollen AIT product profiles for calculating preference shares

Attribute	Pollen Immunotherapy Product Profiles		
	Pre-seasonal SCIT not containing aluminium	Perennial SCIT containing aluminium	SLIT
Product type	An injection, not containing aluminium, taken in the upper arm	Injection, containing aluminium, taken in the upper arm	A tablet placed under the tongue for at least 1 min, and then swallowed as it dissolves
Initial dosing schedule	6 times a year for 3 consecutive years at doctor's office or clinic/hospital before pollen season starts	Monthly for 3 consecutive years at doctor's office or clinic/hospital	Everyday for 3 years taken at home
Side effects	Pain and/or swelling in the mouth/throat in arm	Lumps at injection site (may persist for months/years and can be tender/itchy/have a bad appearance)	Pain and/or swelling in the in arm
Potential future risk^a^	None	Accumulation of foreign material/substance in the body	None

a Affecting a small percentage of patients.

**Table 4:** VIT product profiles for calculating preference shares

Attribute	Venom Immunotherapy Product Profiles		
	aqueous preparation, rush up-dosing	aqueous preparation, conventional up-dosing	depot preparation, conventional up-dosing
Product type	An injection not containing aluminium, taken in the upper arm	An injection not containing aluminium, taken in the upper arm	An injection containing aluminium, taken in the upper arm
Initial dosing schedule	Administered for 2, 3, or 5 consecutive days requiring surveillance in hospital *After initial dose schedule, dosing is monthly for at least 3 years, throughout the year at hospital/clinic or doctor's office*	Administered weekly for 15–16 weeks by a healthcare professional *After initial dose schedule, dosing is monthly for at least 3 years, throughout the year at hospital/clinic or doctor's office*	Administered weekly for 15–16 weeks by a healthcare professional *After initial dose schedule, dosing is monthly for at least 3 years, throughout the year at hospital/clinic or doctor's office*
Side effects	Pain and/or swelling in arm	Pain and/or swelling in arm	Lumps at injection site (may persist for months/years and can be tender/itchy/have a bad appearance)^1^, ^2^, ^3^, ^4^, ^5^, ^6^
Potential future risk^a^	None	None	Accumulation of foreign material/substance in the body

a Affecting a small percentage of patients.

### Statistical analysis

Before statistical analysis was performed, the data was validated and checked for consistency. Survey results were excluded from the analysis when the survey was only answered partially and when it was finished in less than 180s. Respondents were also removed for repetitive answers in the conjoint exercise (see Fig. 1).

Two conjoint analyses were performed to analyze the results of the DCE. A multinomial logit choice model was estimated using the LatentGold Choice 6.0 software.^42^ By default, Wald statistics are provided in the output to assess the statistical significance of the set of parameter estimates associated with a given variable (across all classes). Specifically, for each variable, the Wald statistic tests the restriction that each of the parameter estimates in that set equals zero (for variables specified as Nominal, the set includes parameters for each category of the variable).

The sample size of 700 was considered to receive meaningful results for the pollen cohort. A sample size of >30 participants was considered adequate given the relatively low incidence of venom allergy and the pre-specified inclusion criteria. Due to the overall still small sample sizes, no subgroup analyses were undertaken.

## Results

This section may be divided by subheadings. It should provide a concise and precise description of the experimental results, their interpretation, as well as the experimental conclusions that can be drawn.

### Patients’ characteristics

A total of 943 participants who had an allergy to either pollen (n = 881) or bee and/or wasp stings (n = 62) and had not previously received AIT initially started the survey. After the exclusion of incomplete surveys and fast responders, data from 750 respondents (700 pollen allergic patients and 50 venom-allergic patients) from 7 European countries (Germany, Switzerland, Austria, Spain, Italy, the United Kingdom, and France) were included in the final analysis (Fig. 1).

Of the final completed surveys, 634 (600 for pollen, 34 for venom) were answered by adult patients (mean age: 40 years) and 116 (100 for pollen, 16 for venom) by caregivers of allergic children/adolescents with a mean age of 11 years (Table 5). Answers from caregivers did not differ significantly from answers by adults and were pooled into the main analysis. Female patients were over-represented in the adult groups, at a slightly higher rate than is expected from over-prevalence of allergic diseases in women.^43^^,^^44^

**Table 5:** Participant demographics

	Pollen		Venom	
	Adults	Children	Adults	Children
**Number**	600	100	34	16
**Age in years, mean (SD; min-max)**	39.1 (9.6; 18–55)	11.3 (4.4; 1–18)	39.7 (9.8; 18–54)	8.8 (4.2; 2–15)
**Gender, n (%)**				
Male	192 (32%)	59 (59%)	7 (21%)	10 (63%)
Female	405 (68%)	40 (40%)	27 (79%)	6 (37%)
Other	3 (1%)	1 (1%)	0 (0%)	0 (0%)
**Age of first allergic symptoms**^a^**, mean (SD)**	19.5 (11.1)	6.9 (3.3)	16.5 (13.0)	5.6 (3.8)
**Allergy severity, n (%)**				
Pollen: Mild	349 (59%)	63 (63%)		
Pollen: Moderate/severe	247 (41%)	37 (37%)		
Venom: Grade I^b^			15 (44%)	3 (19%)
Venom: Grade II^b^			9 (27%)	10 (62%)
Venom: Grade III^b^			10 (29%)	3 (19%)
Venom: Grade IV^b^			0 (0%)	0 (0%)

a To pollen or hymenoptera venom respectively.

b According to Mueller et al.

### Conjoint analysis — pollen

**Fig. 2:**
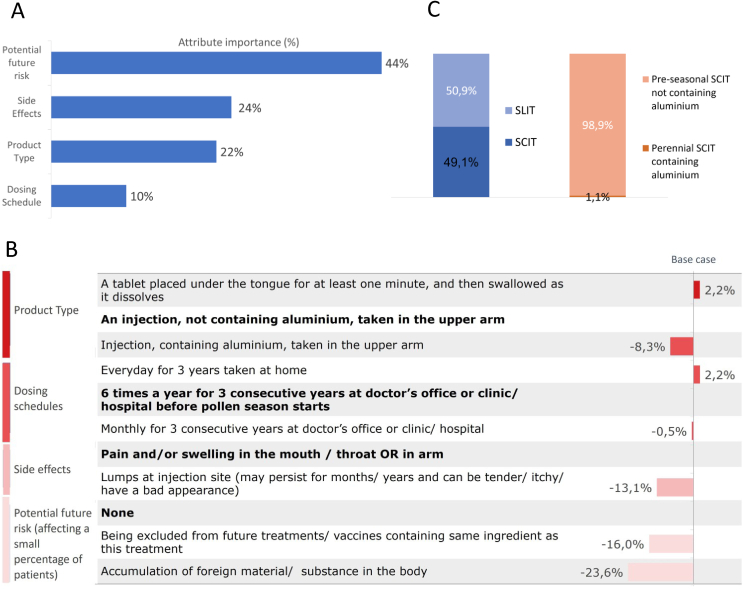
**(A)** Attribute importance pollen AIT. The relative importance (importance score out of 100) of pre-defined attributes against arbitrary base case (in bold print) was highest for the attribute ‘potential future risk’ with 44%, followed by ‘side effects’ (24%), ‘product type’ (22%), and ‘dosing schedule’ (10%). **(B)** Attribute sensitivity pollen AIT. Influence of pre-defined attribute levels on patient preference. The comparator level is written in bold letters. As example: For the attribute ‘side effects’ 2 levels were defined: lumps at the injection site led to a drop in pa-tient preference of −13.1% compared to pain and/or swelling in the arm. **(C)** Patients' preference shares pollen AIT. The calculated preference shares (in%) of patients for predefined pollen AIT product profiles. SLIT received 50.9% of shares compared to SCIT (49.1%). A pre-seasonal, aluminium-free SCIT received 98.9% of shares compared to a perennial SCIT containing aluminium

When calculating the attribute importance (relative importance of defined attributes), potential future risks was the most important attribute for patients when choosing a pollen allergy treatment, with 44% (importance score out of 100). Side effects (24%) and product type (22%) were of moderate importance, while dosing posology appears less important (10%) (Fig. 2 A).

For the attribute “potential future risk”, preferences were negatively impacted by “accumulation of foreign material/substance in the body” by −23.6% (−1.201, p < 0.001) and “being excluded from future treatments/vaccines containing the same ingredient as this treatment” by −16.0% (−0.198, p < 0.001). Patients preferred products with transient side effects like local pain and/or swelling over persistent lumps at the injection site by 13.1% (−0.641, p < 0.001), and sublingual tablets or aluminium-free injections over injections containing aluminium (−8.3%, −0.481, p < 0.001). Dosing schedules were of less importance, with daily SLIT (+2.2%), perennial SCIT (−0.5%) and pre-seasonal SCIT ( ± 0.0%) close in preference (Fig. 2 B). Overall, preference share between SCIT and SLIT product profiles (see Table 3) were 49.1% and 50.9%, respectively (Fig. 2 C). Looking only at SCIT, 98.9% of patients would prefer a pre-seasonal, aluminium-free product over a perennial treatment containing aluminium (Fig. 2 C).

### Conjoint analysis - venom

**Fig. 3:**
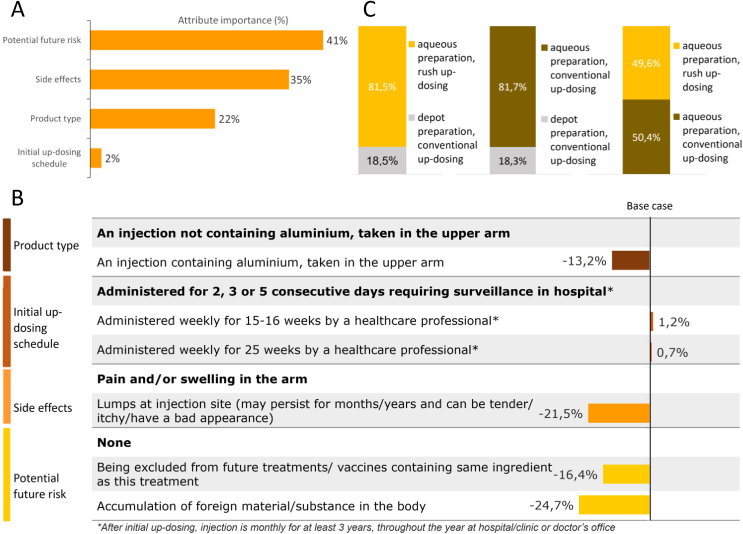
**(A)** Attribute importance VIT. The relative importance (importance score out of 100) of pre-defined attributes against arbitrary base case (in bold print) was highest for the attribute ‘potential future risk’ with 41%, followed by ‘side effects’ (35%), ‘product type’ (22%), and ‘initial up-dosing schedule’ (2%). **(B)** Attribute sensitivity VIT: Influ-ence of pre-defined attribute levels on patient preference. The comparator level is written in bold letters. As exam-ple: For the attribute ‘side effects’ 2 levels were defined: lumps at the injection site led to a drop in patient pref-erence of −21.5% compared to pain and/or swelling in the arm. **(C)** Patient's preference shares VIT. The calculated preference shares (in%) of patients for predefined VIT product profiles. The aqueous preparation with a rush up-dosing posology product profile received 81.5% of shares compared to a depot preparation with conventional up-dosing (18.5%). The aqueous preparation with a conventional up-dosing posology received 81.7% of shares compared to a depot preparation with conventional up-dosing (18.3%). The aqueous preparation with a rush up-dosing posology received 49.6% of shares compared to an aqueous preparation with a conventional up-dosing posology (50.4%)

Similar to the findings for pollen allergy, potential future risks and side effects were the most important attributes for patients when choosing a venom treatment, with 41% and 35%, respectively (importance score out of 100). Less important for the patient was the product type (22%), whereas the initial up-dosing posology seemed not important at all (2%) (Fig. 3 A).

Looking into the sensitivity analysis for the attribute ‘potential future risk’, accumulation of foreign material/substance in the body and being excluded from future treatments/vaccines (containing the same adjuvant as the VIT) revealed the largest negative impact, representing drops in patient preference of −24.7% (−0.54, p < 0.001) and −16.4% (−0.18, p < 0.001), respectively compared to a product with no potential future risks. In addition, lumps at the injection site (that may persist for months or years and can be tender, itchy, and/or have a bad appearance) led to a decline in patient preference of −21.5% (−0.56, p < 0.001) compared to arm pain and/or swelling within in the attribute ‘side effects’. With regards to ‘product type’, injections containing aluminium had a negative impact on the preference for choosing a product of −13.2% (−0.38, p < 0.001) compared to a product without aluminium (Fig. 3 B).

The patients' calculated preference shares for pre-defined VIT product profiles (Fig. 3C, Table 4) clearly favored an aqueous preparation with a rush up-dosing posology. This profile received 81.5% of the shares compared to a depot preparation with conventional up-dosing (18.5%) (Fig. 3 C). Also, when comparing an aqueous with a depot preparation, both with a conventional 15–16 week up-dosing posology, most patients preferred an aqueous product (81.7%) over a depot product (18.3%) (Fig. 3 C). There was only very little difference between the preference shares of the 2 theoretical aqueous product profiles (49.6% vs. 50.4%) (Fig. 3 C), indicating that the initial up-dosing schedule had almost no impact on patient preference for a VIT product profile.

## Discussion

Studies show a positive association between patient preference and treatment adherence and persistence.^3^^,^^4^ As long-term adherence is of preeminent importance for the success of any allergen immunotherapy,^7^ consideration of patient preference is a necessary step in treatment selection. Shared decision making has recently been gaining more recognition in other allergic diseases, with importance placed on consideration of patient preference and values.^45^ This study could aid healthcare professionals to bridge the gap in perspective between patient and caregiver. In fact, in times of the Patients' Rights Act,^46^ physicians should remain humble assuming to know the patient's preference without asking the patient.

In this study, we explored product-related patient preference in AIT in the context of pollen and venom allergy. The data do not show much difference in patient preference with regards to AIT dosing schedules, with perennial or pre-seasonal pollen SCIT, daily pollen SLIT, and various VIT up-dosing schedules perceived with relative indifference. Details like this appear of much greater importance to treating healthcare professionals, for whom the technicalities of administration are the major point of contact with different product options.^47^ Awareness of this disconnect in attributed importance may be critical for effective patient involvement in treatment choice.

Similarly, the decision between SCIT and SLIT, eagerly discussed among healthcare professionals,^48^ does not appear to have a clear answer when patients are asked: Our experiment did not show a clear preference for either form of pollen AIT, with patients being evenly split between both options. This implies that both should be offered to the patient to best address their possible preference.

More importance is placed by the patients on the safety profile of the products involved. Since systemic reactions to AIT are still considered rare for both pollen^10^ and venom^16^ AIT - independent of formulation^15^^,^^49^ - differentiation between products mainly relates to local reactions. Here, the possibility of persistent granuloma represents a major drop in preference in both pollen and venom immunotherapy. Even though aluminium granulomata are considered rare,^10^ the frequencies reported in literature (∼1.0% of patients in a recent prospective vaccination study^50^) imply a high relevance for patients set to receive 30–60 injections over the entire course of their SCIT treatment, potentially even more pronounced in VIT.

This concern is further reflected in the high importance placed on potential future risk and aluminium content of the product. While aluminium is still considered safe for human use by relevant authorities, concerns over the negative impact of accumulation remain 51. These concerns are necessarily amplified in patients who receive regular aluminium doses over long periods of time, ie, patients receiving VIT or perennial pollen SCIT. Some individuals even require both wasp and bee VIT or life-long administration. The difficulties in patient acceptance relating to aluminium raise the question of whether a depot adjuvant is at all necessary, at least in VIT,^49^ as has been posed in other publications.^52^

The high importance placed by allergic patients on the topic of manifest or potential risk of their treatment should be acknowledged by their treating physicians. Discussing the risks associated with each treatment option as an important part of the joint treatment decision may help address patient concerns that would otherwise go unmentioned. This could be facilitated by a structured approach to treatment decision discussions, including decision aids or preference questionnaires.

There are several possible limitations to the study design. For one, the size of the VIT cohort is relatively small, limiting its informative value despite findings being very similar to the larger pollen AIT cohort. The different product attributes have been chosen somewhat arbitrarily based on typical market-available AIT products. While the authors are convinced that the attributes selected are the most relevant for AIT patients, some others (such as evidence levels, allergomics, manufacturing or more detailed posologies) are at least conceivable options and could have affected the outcomes. The exact wording of attributes and accompanying information also may have some influence on the subject's choice.

Inherent biases of the participants or their treating physicians will always be part of patient preference during this study as well as in real-world clinical settings, even though they could be addressed by thorough patient information and education. As an example, the overrepresentation of female patients in the adult groups may introduce a bias towards a higher level of general health-consciousness in the responses, as might the recruitment from a pre-existing pool of patients open to answering health-related questions. Also, while this study establishes data on patient preference on abstract, theoretical attributes, real life preferences may change in patients experiencing AIT. Attributes like dosing schedules and treatment duration could become more important to patients once they need to adapt their day-to-day schedules to their treatment. Therefore, preferences expressed in this experiment based on hypothetical scenarios may potentially differ from real-world choices once the patient actually chooses or starts their treatment. The study also needs to account for potential innovations in AIT. While current dosing schedules have little influence on patient preference, and available depot formulations are met with strong disapproval from patients, future options with more patient-friendly dosing schedules or different adjuvants, for examples, could change these findings.

Finally, the actual effect of shared decision making and consideration of patient preference on real-world adherence specifically in AIT has so far not been explored; further research on such effects is warranted, and may help in guiding evaluation and recommendation of AIT treatment options for regulators and guidelines.

## Conclusions

Thorough involvement of an informed patient in the treatment decision process (= shared decision-making), is an often-overlooked enabler for adherence and persistence. Concerns about potential future risks and side effects mainly drive patient preference for being treated with a particular venom immunotherapy, therefore physicians should clearly communicate all available information with the patient. The preference between SLIT and SCIT for pollen allergy treatment is split evenly, and more than 80% of patients would prefer an AIT product without aluminium – independent of posology. The results of this study warrant further research, especially into the real-world influence of patient preference and its consideration on adherence in AIT.

## Abbreviations

AIT, Allergen Immunotherapy; CBC, Choice-Based Conjoint (software by Sawtooth); DCE, Discrete Choice Experiment; DEGS1, German Health Interview and Examination Survey for Adults; EAACI, European Academy of Allergy and Clinical Immunology; EphMRA, European Pharmaceutical Market Research Association; ESOMAR, European Society for Opinion and Marketing Research; IQWiG, Institute for Quality and Efficiency in Health Care (Germany); ISPOR, International Society for Pharmacoeconomics and Outcomes Research; MCT, MicroCrystalline Tyrosine; MPL, Monophosphoryl Lipid-A; MRS, Market Research Society; PEI, Paul-Ehrlich-Institute; SCIT, Subcutaneous Immunotherapy; SDM, Shared Decision-Making; SLIT, Sublingual Immunotherapy; UK, United Kingdom

## Author's consent for publication

All authors have approved the manuscript and agree with its submission to World Allergy Organization.

## Confirmation of unpublished work

This manuscript is original, has not been published before, and is not currently being considered for publication elsewhere.

## Informed consent/ethics statement

Participation in the online survey was voluntary and could be withdrawn at any time. European Data Protection Law was followed, and only anonymized aggregated results were provided, therefore patient consent was waived.

## Author contributions

S. Becker: data analysis and interpretation, writing of original manuscript draft, review and editing of manuscript, data visualization. M. Feindor, A. Graessel, K. Birkholz, J. Raab, D. Hernandez, M.F. Kramer: data analysis, data interpretation and visualization, writing of original manuscript draft. I. Fernández de Alba: data analysis, data interpretation, writing of original manuscript draft. G. Hofer: Conceptualization, project administration, data analysis. R. Abro: review and editing of manuscript. N. Valentine: Survey methodology, conduct of survey on behalf of the market research fieldwork panel provider Lumanity, data collection and analysis, review and editing of manuscript. F. Fassio, O. Fuchs, S. Blank, L. Klimek, E. Jensen-Jarolim, M. Ollert, D. Gonzalez-de-Olano, P. Schmid-Grendelmeier: data interpretation, review and editing of manuscript. All authors have read and approved the submitted version of the manuscript.

## Data availability

The data that support the findings of this study are not publicly available, but are available from the corresponding author upon reasonable request.

## Declaration of Generative AI and AI-assisted technologies in the writing process

Nothing to disclose.

## Funding

This research was funded by 10.13039/100017255Allergy Therapeutics, Worthing, UK.

## Competing interests

S. Becker reports grants from Bencard Allergie, BRAIN AG, Karl Storz GmbH, Altamira AG and the German Federal Ministry of Education and Research. Honoraria for Advisory Boards and presentations from Allergy Therapeutics, Bencard Allergie, HAL Allergy, Allergo-pharma, ALK Abelló, Sanofi, Novartis, GSK, AstraZeneca, MSD, Viatris, Ambu and Stryker, out-side the submitted work. N. Valentine is an employee of Lumanity. F. Fassio received consultancy fees from Allergy Therapeutics Italia srl, outside the submitted work. S. Blank has given advice or has received honorariums for talks or research grants from Thermo Fischer Scientific Inc., Bencard Allergie GmbH, Allergopharma GmbH & Co. KG, LETI Pharma GmbH, and Allergy Therapeutics PLC, outside the submitted work. E. Jensen-Jarolim received grants from Danube Allergy Research Cluster, Karl Landsteiner University; earns royalties/holds licenses EP 2894478 A1, owned by Bi-omedical Int. R + D GmbH, Vienna, Austria; LK reports grants and personal fees from Allergo-pharma, grants and personal fees from MEDA/Mylan, personal fees from HAL Allergie, grants from ALK Abelló, grants and personal fees from LETI Pharma, grants from Stallergenes, grants from Quintiles, grants and personal fees from Sanofi, grants from ASIT biotech, grants from Lofarma, personal fees from Allergy Therapeut., grants from AstraZeneca, grants from GSK, grants from Inmunotek, personal fees from Cassella med, outside the submitted work; and Membership: AeDA, DGHNO, Deutsche Akademie für Allergologie und klinische Immunologie, HNO-BV, GPA, EAACI. EJJ is shareholder of Biomedical Int. R + D GmbH, Vienna, Austria; received payment or honoraria for lectures and presentations from Bencard Allergie GmbH, Allergy Eherapeutics Ltd, Sanofi, Santen, Novartis, Menarini, GSK; owns patent EP 2894478 A1, participated on Data Safety Monitoring Board or Advisory Board for Bencard Allergie GmbH, Allergy Therapeutics, is the President and past president of Austrian Society of Allergy and Immunology; is shareholder in Biomedical Int. R + D GmbH, Vienna, Austria; all outside the submitted work. M. Ollert reports honoraria for Advisory Boards and presentations from Allergy Therapeutics/Bencard Allergie and Hycor Diagnostics, and grant support from Luxembourg National Research Fund FNR, European Commission Horizon Europe, outside the submitted work. I. Fernández de Alba, O. Fuchs, D. Gonzalez-de-Olano and P. Schmid-Grendelmeier report no conflict of interest in relation to this publication. M. Feindor, K. Birkholz, J. Raab, R. Abro, A. Graessel, D. Hernandez, G. Hofer and M.F. Kramer are employees of Allergy Therapeutics/Allergy Therapeutics Ibérica/Bencard Allergie GmbH, a company using biodegradable adjuvants.
