# Supplementary material for: Patient preference in allergen immunotherapy - Understanding the patient's view
Source: World Allergy Organ J. 2025 Dec 13;18(12):101154. doi: 10.1016/j.waojou.2025.101154 (PMC12765409; doi:10.1016/j.waojou.2025.101154)
Supplement: Multimedia component 1 [file mmc1.pdf]

Supplemental Material

Becker S. et al., Patient Preference in Allergen Immunotherapy - Understanding the Patient's View

H0. AUTOFILL

Single code

Country

1. Germany – **ACCEPT**
2. Austria – **ACCEPT**
3. Switzerland – **ACCEPT**
4. Spain – **ACCEPT**
5. France – **ACCEPT FOR VENOM ONLY**
6. UK – **ACCEPT FOR VENOM ONLY**
7. Italy – **ACCEPT FOR VENOM ONLY**
8. Other – **SCREEN OUT**

Qintro1 – ASK ALL

Single code

Thank you for agreeing to take part in this survey. Please use the [INSERT >> OR NEXT AS APPROPRIATE] below to navigate through this questionnaire. Using the back button on your browser may cause you to exit the survey before the end.

The survey is being conducted by Lumanity, an independent market research agency, on behalf of a pharmaceutical company, and we are interested in speaking with people like yourself across several countries.

The purpose of this research is to understand more about your health situation, health issues and treatment.

Please be assured that we act in accordance with the EphMRA (European Pharmaceutical Market Research Association), ESOMAR (European Society for Opinion and Marketing Research) and MRS (Market Research Society) codes of conduct regarding anonymity and confidentiality. We also abide by the Data Protection Act.

We comply with the European Data Protection Law. Your privacy will be protected at all times unless you give specific permission to be identified. The personal data you provided when signing up to the panel (e.g. name, phone number, and address) is stored by the fieldwork agency that invited you to this study. Full details of how they protect your data are available in their privacy policy which you can find in the email invitation you received from them.]

Participation is entirely voluntary and you can withdraw from the survey at any time. Your privacy will be protected at all times unless you give specific permission to be identified.

Firstly, we need to ask you some questions to ensure that the market research study is appropriate for you.

By entering the survey link you confirm that you understand and agree to the following:

1. This research is sponsored by another company and is being carried out within the Market Research codes of conduct
2. This research will collect your views for market research purposes only and is not promotional
3. Taking part is completely voluntary and you have the right to withdraw from the survey at any time and to withhold information as you see fit. To withdraw from the survey, exit the browser at any time
4. Your identity will remain confidential and none of your personal information will be shared with any 3rd party
5. The anonymized aggregated results of the study may be used for Marketing or PR purposes or scientific publications. In such cases the identity of each participant in the survey remains confidential and no details of any individual are passed to any 3rd party
6. You may, at any time, ask to know what personal data we hold about you and subsequently request your personal details to be amended or destroyed. Please find our privacy policy link here: https://lumanity.com/privacy-policy/
7. Anything you see or read during this research should be treated as confidential

Please select one option only.

1. I agree to all of these statements
2. I do not agree with at least one of these statements **SCREEN OUT**

Qintro4 – ASK RESPONDENTS IN AUSTRIA / SWITZERLAND

Single code

Do you feel comfortable reading, writing and speaking German?

Please select one option only.

1. Yes
2. No **SCREEN OUT**

SCREENER

Qintro2 - TEXT

Firstly, we need to ask you some questions to ensure that the market research study is appropriate for you.

S1. ASK ALL <MULTI CODE> RANDOMISE

Which of the following conditions do you suffer from?

1. Allergies
2. Migraines
3. Psoriasis
4. Shingles
5. Diabetes
6. Asthma
7. None of the above **– EXCLUSIVE, BUT PROCEED TO S1b**

IF CODE 1 SELECTED, AUTOCODE AS ADULT PATIENT

S1b. ASK ALL <MULTI CODE> RANDOMISE

Which of the following conditions does the child you care for suffer from?

By child you care for, we mean anyone under the age of 18 years in your household that you have responsibility for.

1. Allergies
2. Migraines
3. Psoriasis
4. Shingles
5. Diabetes
6. Asthma
7. None of the above EXCLUSIVE

IF CODE 1 SELECTED, AUTOCODE AS CARETAKER OF PAEDIATRIC PATIENT

IF NEITHER ADULT PATIENT NOR A CARETAKER OF PAEDEATRIC PATIENT TERMINATE

S2. ASK IF ADULT PATIENT (CODE 1 SELECTED AT S1) <MULTI CODE> KEEP RESPIRATORY ALLERGIES UNDER THE HEADER BUT RANDOMISE WITHIN THESE ALLERGIES

Which allergies do you suffer from?

Respiratory allergy

Pollen allergy, including grass, tree, ragweed or other pollens
House dust mite allergy
Animal dander allergy
Mold allergy

[NOTE FOR SCRIPTERS: The following are not respiratory allergies]
Allergy to bee / wasp stings
Food allergy (peanut or other food)
Latex allergy
Other

IF CODE 02, ADULT PATIENT POLLEN QUOTA
IF CODE 01 ADULT PATIENT VENOM QUOTA

S2b. ASK IF CARETAKER OF PAEDIATRIC PATIENT (CODE 1 SELECTED AT S1B) <MULTI CODE> KEEP RESPIRATORY ALLERGIES UNDER THE HEADER BUT RANDOMISE WITHIN THESE ALLERGIES

Which allergies does the child you care for suffer from?

Respiratory allergy

Pollen allergy, including grass, tree, ragweed or other pollens
House dust mite allergy
Animal dander allergy
Mold allergy

[NOTE FOR SCRIPTERS: The following are not respiratory allergies]
Allergy to bee / wasp stings
Food allergy (peanut or other food)
Latex allergy
Other

IF CODES 01, 02 NOT SELECTED IN BOTH S2 OR S2b, TERMINATE
IF CODE 01 CARETAKER OF PAEDIATRIC PATIENT VENOM QUOTA
IF CODE 02, CARETAKER OF PAEDIATRIC PATIENT POLLEN QUOTA

IF CODE 01 (bee /wasp sting allergy) NOT SELECTED IN UK, FR, IT:
IF ONLY ADULT QUOTA SO FAR, TERMINATE HERE IF CODE 01 NOT SELECTED
IF ONLY CAREGIVER QUOTA SO FAR, TERMINATE HERE IF CODE 01 NOT SELECTED
IF QUALIFY FOR BOTH SO FAR, TERMINATE HERE IF CODE 01 NOT SELECTED FOR EITHER

IF RESPONDENT QUALIFIES ON BOTH S2 AND S2b AS ADULT PATIENT AND A CARETAKER OF PAEDEATRIC PATIENT, FILL QUOTA BASED ON DEVELOPMENT IN

FIELD, AND CODE THEM ACORDINGLY. ALWAYS PRIORITISE BEE/ WASP STING ALLERGY QUALIFICATION FILL QUOTA AFTER S7

S3. ASK IF ADULT PATIENTS <MULTI CODE>

Which of your allergies have been confirmed or diagnosed by a doctor?

1. **LIST ALLERGIES SELECTED AT S2**
2. None of the above
   THANK AND CLOSE IF POLLEN ALLERGY NOT CODED FOR POLLEN ALLERGY QUOTA (NO TERMINATION FOR VENOM ALLERGY)

S3b. ASK IF CARETAKER OF PAEDIATRIC PATIENT <MULTI CODE>

Which of your child's allergies have been confirmed or diagnosed by a doctor?

1. **LIST ALLERGIES SELECTED AT S2b**
2. None of the above
   THANK AND CLOSE IF POLLEN ALLERGY NOT CODED FOR POLLEN ALLERGY QUOTA (NO TERMINATION FOR VENOM ALLERGY)

S4. ASK IF ADULT PATIENTS < SINGLE CODE>

Have you ever been treated with allergen immunotherapy? This is also known as hyposensitisation or desensitisation, an allergy treatment designed to treat the underlying cause of the disease and which can have a long-lasting effect to reduce or eliminate your allergy altogether. Immunotherapy involves exposing people to larger and larger amounts of allergen in an attempt to change the immune system's response

1. Yes
2. No

   THANK AND CLOSE IF YES

S4b. ASK IF CARETAKER OF PAEDIATRIC PATIENT < SINGLE CODE>

Has the child you care for ever been treated with allergen immunotherapy? This is also known as hyposensitisation or desensitisation, an allergy treatment designed to treat the underlying cause of the disease and which can have a long-lasting effect to reduce or eliminate your allergy altogether. Immunotherapy involves exposing people to larger and larger amounts of allergen in an attempt to change the immune system's response

1. Yes

2. No

THANK AND CLOSE IF YES

S5. ASK ALL <MULTI CODE>

Are you or an immediate family member currently employed by or under contract with any of the following?

1. Manufacturer of allergy-related products – THANK AND CLOSE
2. A marketing or market research department or company – THANK AND CLOSE
3. An advertising agency – THANK AND CLOSE
4. A pharmaceutical company or healthcare manufacturer – THANK AND CLOSE
5. None of the above

S6. ASK ALL <Numeric, Range 1-110>

How old are you?

Please enter your age in the box.

1. ______ years

THANK AND CLOSE IF LESS THAN 18 OR GREATER THAN 55 AND QUALIFIED AS ADULT PATIENT

IF CARETAKER HAVE TO BE 18 OR GREATER

S7. ASK CARETAKER OF PAEDIATRIC PATIENT <Numeric, Range 1-110>

How old is the child you care for?

Please enter age in the box.

1. ______ years

THANK AND CLOSE IF 18+

RECRUIT A MIX OF AGES WITHIN ADULT AND WITHIN PAEDIATRIC QUOTAS

S8. ASK <u>ADULT PATIENTS</u> <Single code>

What is your gender?

Please select one option only.

1. Male
2. Female
3. Other
4. Prefer not to say

RECRUIT A MIX

S9. ASK <u>CARETAKER OF PAEDIATRIC PATIENT</u> <Single code>

What is the gender of the child that you care for?

Please select one option only.

1. Male
2. Female
3. Other
4. Prefer not to say

RECRUIT A MIX

IF RESPONDENT QUALIFIES AS ADULT PATIENT AND A CARETAKER OF PAEDEATRIC PATIENT, FILL QUOTA BASED ON DEVELOPMENT IN FIELD, AND CODE THEM ACORDINGLY. ALWAYS PRIORITISE BEE/ WASP STING ALLERGY QUALIFICATION

Qintro3. TEXT – Show all who qualify

Thank you. You are eligible to participate in the research. Please click the arrow to continue.

Screenout1. TEXT – Show all who Screen out

Thank you for your willingness to participate. Unfortunately, you do not qualify for this particular study at this time. We hope you will consider us for future studies.

Overquota. TEXT – Show all who are overquota

Thank you for your willingness to participate. Unfortunately the total number of participants for this study is currently met. We will re-contact you if the survey re-opens.

CONJOINT EVALUATION

Qintro4 SHOW ALL

POLLEN ALLERGY ADULT PATIENTS - Thanks for your answers so far. The following questions relate to the pollen allergy that you have.

POLLEN ALLERGY CARETAKES OF PAEDIATRIC PATIENTS - Thanks for your answers so far. The following questions relate to the pollen allergy that the child that you care for has.

VENOM ALLERGY ADULT PATIENTS - Thanks for your answers so far. The following questions relate to the bee / wasp sting allergy that you have.

POLLEN ALLERGY CARETAKES OF PAEDIATRIC PATIENTS - Thanks for your answers so far. The following questions relate to the bee / wasp allergy that the child that you care for has.

Q1. SHOW ALL POLLEN ALLERGY ADULT PATIENTS AND CARETAKERS OF PAEDIATRIC PATIENTS

In this next section, we will be sharing some potential treatment options for a new medication for the treatment of **pollen** allergy, Product X. This medication is an Allergen Immunotherapy.

Allergen Immunotherapy (AIT), also known as hyposensitisation or desensitisation, is an allergy treatment designed to treat the underlying cause of the disease and which can have a long-lasting effect to reduce or eliminate your allergy altogether.

In the following exercise, you will see versions of Product X, a new medication for the treatment of **pollen** allergy, with some variations in characteristics.

You will see a total of **6 screens** for this section.

On each screen, please look at the **3** potential treatment options, and indicate what treatment you would choose [ADULT PATIENTS for your pollen allergy] [CARETAKERS OF PAEDIATRIC PATIENTS for the pollen allergy of the child you care for], if these versions of Product X are available on the market. Please select **one** treatment only.

When you are ready to see the first screen please select 'Continue'.

SHOW SCENARIOS BASED ON CONJOINT DESIGN

ON TOP OF EACH SCREEN SHOW MESSAGE:

Which one of these treatments would you choose [ADULT PATIENTS for your pollen allergy] [CARETAKERS OF PAEDIATRIC PATIENTS for the pollen allergy of the child you care for]? Please select **one** treatment only. Please look through the choices carefully as they have some differences between them.

INPUTS TO CONJOINT DESIGN POLLEN

ATTRIBUTE	LEVELS			
	1	2	3	4

**Product type**	A tablet placed under the tongue for at least one minute, and then swallowed as it dissolves	An injection, not containing aluminium, taken in the upper arm	Injection, containing aluminium, taken in the upper arm	
**Initial dosing schedule**	Everyday for 3 years taken at home	6 times a year for 3 consecutive years at doctor's office or clinic/ hospital before pollen season starts	Monthly for 3 consecutive years at doctor's office or clinic/ hospital	
**Side effects**	Pain and/or swelling in the mouth / throat **[SHOW IF TABLET]**/ in arm **[SHOW IF INJECTION]**	Lumps at injection site (may persist for months/ years and can be tender/ itchy/ have a bad appearance)		
**Potential future risk (affecting a small percentage of patients)**	None	Being excluded from future treatments/ vaccines containing same ingredient as this treatment	Accumulation of foreign material/ substance in the body	

Q2. SHOW ALL VENOM ALLERGY ADULT PATIENTS AND CARETAKERS OF PAEDIATRIC PATIENTS

In this next section, we will be sharing some potential treatment options for a new medication for the treatment of **bee/wasp sting** allergy, Product X. This medication is an Allergen Immunotherapy.

Allergen Immunotherapy (AIT), also known as hyposensitisation or desensitisation, is an allergy treatment designed to treat the underlying cause of the disease and which can have a long-lasting effect to reduce or eliminate your allergy altogether.

In the following exercise, you will see versions of Product X, a new medication for the treatment of **bee/wasp sting** allergy, with some variations in characteristics.

You will see a total of **12 screens** for this section.

On each screen, please look at the **3** potential treatment options, and indicate what treatment you would choose [ADULT PATIENTS for your bee/wasp sting allergy] [CARETAKERS OF PAEDIATRIC PATIENTS for the bee/wasp sting allergy of the child you care for], if these versions of Product X are

on the market and available. Please select **one** treatment only. Please look through the choices carefully as they have some differences between them.

When you are ready to see the first screen please select 'Continue'.

SHOW SCENARIOS BASED ON CONJOINT DESIGN

ON TOP OF EACH SCREEN SHOW MESSAGE:

Which one of these treatments would you choose [ADULT PATIENTS for your bee/wasp sting allergy] [CARETAKERS OF PAEDIATRIC PATIENTS for the bee/wasp allergy of the child you care for]? Please select **one** treatment only.

INPUTS TO CONJOINT DESIGN BEE/ WASP

**ATTRIBUTE**	**LEVELS**		
	**1**	**2**	**3**
**Product type**	An injection not containing aluminium, taken in the upper arm	An injection containing aluminium, taken in the upper arm	
**Initial dosing schedule**	Administered for 2, 3 or 5 consecutive days requiring surveillance in hospital<br><br>*After initial dose schedule, dosing is monthly for at least 3 years, throughout the year at hospital / clinic or doctor's office*	Administered weekly for 15-16 weeks by a healthcare professional<br><br>*After initial dose schedule, dosing is monthly for at least 3 years, throughout the year at ~~a~~ [hospital / clinic or doctor's office*	Administered weekly for 25 weeks by a healthcare professional<br><br>*After initial dose schedule, dosing is monthly for at least 3 years, throughout the year at ~~a~~ hospital / clinic or doctor's office*
**Side effects**	Pain and/or swelling in arm	Lumps at injection site (may persist for months/ years and can be tender/ itchy/ have a bad appearance)	
**Potential future risk (affecting a small percentage of patients)**	Being excluded from future treatments/ vaccines containing same ingredient as this treatment	Accumulation of foreign material/substance in the body	

DEMOGRAPHICS

D1. ASK ADULT PATIENTS (CODE 1 SELECTED AT S1) <Numeric, Range AGE AT <=S6>

What age were you when you first started experiencing symptoms of [POLLEN ALLERGY: pollen allergy] [VENOM ALLERGY: bee/wasp sting allergy]?

Please enter age in the box.

1. _____ years

D2. ASK CARETAKERS OF PAEDEATRIC PATIENTS (CODE 1 SELECTED AT S1B) <Numeric, Range AGE <=S7>

What age was the child you care for when they started experiencing symptoms of [POLLEN ALLERGY: pollen allergy] [VENOM ALLERGY: bee/wasp sting allergy]?

Please enter age in the box.

1. _____ years

Q3. ASK POLLEN ALLERGY <MULTI CODE> RANDOMISE

[ADULT PATIENTS WITH POLLEN ALLERGY] Which symptoms do you experience as a result of your pollen allergy?

[CARETAKERS OF PAEDEATRIC PATIENTS WITH POLLEN ALLERGY] Which symptoms does the child you care for experience as a result of their pollen allergy?

1. Nasal congestion
2. Sinus pressure/facial pain
3. Runny nose
4. Itchy, watery eyes
5. Sore throat
6. Decreased sense of taste or smell
7. Asthmatic reactions / Increased asthmatic reactions
8. Oral allergy syndrome – itchiness in mouth and throat immediately after eating fresh fruit or vegetables
9. Other (please specify)

Q4. ASK VENOM ALLERGY <MULTI CODE> RANDOMISE

[ADULT PATIENTS WITH VENOM ALLERGY] Which symptoms do you experience as a result of your bee/wasp sting allergy?

[CARETAKERS OF PAEDIATRIC PATIENTS WITH VENOM ALLERGY] Which symptoms does the child you care for experience as a result of their bee/wasp allergy?

1. Hives
2. Itching
3. Flushed or pale skin
4. Difficulty breathing
5. Swelling of the lips, tongue and throat
6. A weak or rapid pulse
7. Decrease in blood pressure
8. Nausea, vomiting or diarrhoea
9. Abdominal cramping
10. Dizziness or fainting
11. Loss of consciousness
12. Other (please specify)

Q5a. ASK IF CODED 1-4 IN Q3 <SINGLE CODE>

[ADULT PATIENTS WITH POLLEN ALLERGY] How would you describe the severity of your allergic symptoms (nasal congestion, sinus pressure/ facial pain, runny nose, itchy watery eyes) due to your pollen allergy?

[CARETAKERS OF PAEDIATRIC PATIENTS WITH POLLEN ALLERGY] How would you describe the severity of the allergic symptoms (nasal congestion, sinus pressure/ facial pain, runny nose, itchy watery eyes) due to the pollen allergy of the child you care for?

1. Mild (normal sleep, normal work or school, normal daily activity, no disturbing symptoms)
2. Moderate/ Severe (one or more of the following – disturbed sleep, problems at work or school, impairment of daily activity or sport, disturbing symptoms)

Q5b. ASK IF CODED 1-4 IN Q3 <SINGLE CODE>

[ADULT PATIENTS WITH POLLEN ALLERGY] How would you describe the frequency of your allergic symptoms (nasal congestion, sinus pressure/ facial pain, runny nose, itchy watery eyes) due to your pollen allergy?

[CARETAKERS OF PAEDIATRIC PATIENTS WITH POLLEN ALLERGY] How would you describe the frequency of the allergic symptoms (nasal congestion, sinus pressure/ facial pain, runny nose, itchy watery eyes) due to the pollen allergy of the child you care for?

1. Intermittent (<4 days/ week or <4 weeks/ episode)

2. Persistent (>4 days/ week and >4 weeks/ episode)

Q5.1. ASK <u>VENOM ALLERGY</u> <SINGLE CODE>

[ADULT PATIENTS WITH VENOM ALLERGY] How would you describe the severity of your bee/wasp sting allergy?

[CARETAKERS OF PAEDIATRIC PATIENTS WITH VENOM ALLERGY] How would you describe the severity of the bee/wasp sting allergy for the child you care for?

1. Grade I- Systemic reaction characterised by generalised urticaria (raised, itchy rash) or erythema (abnormal redness of skin e.g. red splotches), itching, malaise or anxiety.
2. Grade II- may include symptoms associated with grade I reactions plus two or more of the following - generalised oedema (swelling caused by fluid build-up), tightness in the chest, wheezing, abdominal pain, nausea and vomiting, and dizziness
3. Grade III- reactions may include symptoms associated with grade I or II reactions plus two or more of the following - symptoms of dyspnoea (difficult or laboured breathing), dysarthria (difficulty in speaking – slurred, slow speech), hoarseness, weakness, confusion, and a feeling of impending doom
4. Grade IV- Reactions may include symptoms associated with grade I, II or III reactions plus two or more of the following - loss of consciousness, incontinence (lack of voluntary control) of urine or faeces, or cyanosis (poor blood circulation and lack of oxygen in blood causing bluish skin)

Q6. ASK <u>POLLEN ALLERGY</u> <MULTI CODE> RANDOMISE

[ADULT PATIENT, POLLEN ALLERGY] Which treatments have you tried for your pollen allergy?

[CARETAKER OF PAEDIATRIC PATIENT, POLLEN ALLERGY] Which treatments has the child you care for tried for their pollen allergy?

1. Oral antihistamines (i.e. cetirizine (Zyrtec), Fexofenadine (Allegra), Levocetirizine (Xyzal), loratadine (Claritin)) [SPAIN ONLY: desloratadina (Aerius), bilastina (Bilaxten, Ibis), ebastina (Ebastel), rupatadina (Alergoliber, Rinialer, Rupafin).]
2. Oral decongestants (i.e. cetirizine-pseudoephedrine (Zyrtec-D 12 Hour), fexofenadine-pseudoephedrine (Allegra-D 12 Hour Allergy and Congestion), loratadine-pseudoephedrine (Claritin-D)) SPAIN ONLY: cetirizina-pseudoefedrina (Stopcold)]
3. Nasal spray, decongestants (i.e. oxymetazoline hydrochloride ([ALL EXCEPT SPAIN: Afrin, Dristan, Sinex [SPAIN: Respibien, Interfrin, Oximetazolina Farline]) and phenylephrine hydrochloride (ALL EXCEPT SPAIN: Neo-Synephrine) SPAIN: Fenilefrina Altan]) SPAIN ONLY: lebocabastina (Bilina), oximetazolina (Utabon, Respibien, Cuvenax, Respir).
4. Nasal corticosteroid sprays (i.e. beclomethasone (ALL EXCEPT SPAIN: Beconase, Qnasl SPAIN: Becotide, Soprobec), ciclesonide (ALL EXCEPT SPAIN: Zetonna SPAIN: Alvesco), fluticasone furoate (ALL EXCEPT SPAIN: Veramyst SPAIN: Avamys), mometasone (Nasonex), budesonide (Rhinocort Allergy), fluticasone (ALL EXCEPT SPAIN: Flonase Allergy Relief SPAIN: Flonase), triamcinolone (ALL EXCEPT SPAIN: Nasacort Allergy 24HR) SPAIN: Nasacort])
5. Home remedies (herbal teas, saline sprays, nasal rinses, acupuncture)

99. None of the above

Q6.1. ASK VENOM ALLERGY <MULTI CODE> RANDOMISE

[ADULT PATIENT, VENOM ALLERGY] Which treatments have you tried for your bee/wasp sting allergy?

[CARETAKERS OF PAEDIATRIC PATIENT, VENOM ALLERGY] Which treatments has the child you care for tried for their bee/wasp sting allergy?

1. Oral antihistamines (i.e. cetirizine (Zyrtec), Fexofenadine (Allegra), Levocetirizine (Xyzal), loratadine (Claritin)) [SPAIN ONLY: desloratadina (Aerius), bilastina (Bilaxten, Ibis), ebastina (Ebastel), rupatadina (Alergoliber, Rinialer, Rupafin).]
2. Over the counter pain relievers (i.e. acetaminophen (Tylenol) or ibuprofen (Motrin))
3. Steroid cream (i.e. beclomethasone (SPAIN ONLY: Menaderm), betamethasone (SPAIN ONLY: Alergical, Celecrem, Celestoderm, Daivobet), clobetasol (SPAIN ONLY: Colvate, Decloban), fluticasone (SPAIN ONLY: Fluticrem), mometasone (SPAIN ONLY: Elocom), hydrocortisone cream ALL EXCEPT SPAIN AND ITALY: (Cortizone-10 SPAIN ONLY: Lactisona), SPAIN ONLY: fluocinolone (Synalar) ITALY ONLY: Loicodon with Idrocortisone 17-butirrato) GERMANY ONLY THESE EXAMPLES (z. B. Fenistil-Hydrocort, Ecural, Dermatop)
4. Analgesic cream (i.e. benzocaine or lidocaine)
5. Epinephrine (Adrenaclick, Auvi-Q, EpiPen, Symjepi, or a generic version of the auto-injector)
6. IV antihistamines and cortisone
7. Home remedies (cold compress, baking soda)

99. None of the above

Q7.1 ASK ALL <SINGLE CODE>

Which bracket does your annual household income fall in before tax is deducted?

UK

1. £20,000 or below
2. £20,001 – 40,000
3. £40,001 - 60,000
4. £60,001-80,000
5. £80,001-100,000
6. £100,001 -120,000
7. >£120,000

FRANCE/ ITALY/ SPAIN/ GERMANY/ AUSTRIA/SWITZERLAND

1. €20,000 or below
2. €20,001 – 40,000
3. €40,001 - 60,000
4. €60,001-80,000
5. €80,001-100,000
6. €100,001 -120,000
7. >€120,000

CLOSING

Qclose1. ASK ALL «Single code»

Thank you for your answers. Would you be willing to be re-contacted regarding any follow up questions we may have for this survey?

1. Yes
2. No

THANK AND CLOSE
